# Editorial: Pharmacology of L-Arginine and L-Arginine-Rich Food

**DOI:** 10.3389/fphar.2021.743788

**Published:** 2021-08-03

**Authors:** Burkhard Poeggeler, Horst Robenek, Miguel A. Pappolla

**Affiliations:** ^1^ Johann-Friedrich-Blumenbach-Institute for Zoology and Anthropology, Faculty of Biology and Psychology, Georg August University Göttingen, Göttingen and Goettingen Research Campus, Göttingen, Germany; ^2^ Department of Neurology, University of Texas Medical Branch, University Boulevard, Galveston, TX, United States; ^3^ Leibniz-Institut für Arterioskleroseforschung, Universitätsklinikum Münster, Münster, Germany

**Keywords:** ageing, arginine, asymmetric dimethylarginine ADMA, citrulline, lung disease, nitric oxide

The data and findings presented in this article collection on the pharmacology of L-arginine and L-arginine-rich food indicate that approaches aimed at regaining metabolic control and restoring health in the elderly are urgently needed. Amino acids are the building blocks of life that enable repair and regeneration. L-Arginine maintains metabolic regulation, cardiovascular health, blood flow, cognitive performance, muscle mass and body composition by enhancing nitric oxide formation (Curis et al., 2005; El Assar et al., 2016; Jankovic et al., 2017). The molecular master modulators and mechanisms of redox regulation include nitric oxide (NO) and superoxide anion radicals (SOR). Studies on NO have demonstrated anti-ageing effects by improving blood flow, maintaining endothelial health and stimulating mitochondrial activity and biogenesis (Gambardella et al., 2020). Ageing is often associated with increased adiposity, altered and reduced muscle mass or sarcopenia including increased ectopic fat stores some of which are independently associated with increased cardiometabolic risk and physical dysfunction (Curis et al., 2005; El Assar et al., 2016; Jankovic et al., 2017). The development of insulin resistance is associated with endothelial dysfunction, which has been observed in age dependent metabolic diseases such as obesity and diabetes (Curis et al., 2005; El Assar et al., 2016; Jankovic et al., 2017). A reduced NO formation by insufficient supply of L-arginine leads to an endothelial dysfunction that compromises the endogenous supply of energetic resources to enable regeneration (Curis et al., 2005; El Assar et al., 2016; Jankovic et al., 2017). The age dependent increase of asymmetric dimethylarginine levels and the upregulation of arginase activity reduce NO formation and constitute important factors of alterations in the L-arginine/nitric oxide pathway (Curis et al., 2005; Pizzarelli et al., 2013; El Assar et al., 2016; Jankovic et al., 2017; Cziráki et al.; Huang et al.). They may lead to insulin resistance and endothelial dysfunction with impaired vasodilation (Shatanawi et al.; Scott et al.). Such changes promote degenerative processes that can lead to disorders and diseases associated with morbidity and mortality (Pizzarelli et al., 2013; Cziráki et al.; Huang et al.). The current challenge to research is to target inflammaging and explore new approaches that modify L-arginine metabolism and nitric oxide formation specifically and successfully (Cziráki et al.; Shatanawi et al.; Huang et al.; Scott et al.). The long term mono supplementation with high doses of L-arginine can exert detrimental effects (Huang et al.). L-arginine combined with B vitamins like folic acid can be given at much lower doses and folic acid can antagonize the induction of arginase, the depletion of NO and the consequent prooxidant inflammatory reactions (Nikolić et al., 1993; Fatahi et al., 2019; Youssry and Kamel, 2019). The progress in the field depends on the development of a precision supplementation in which the supply of the protective agents is specifically adjusted to the demand (Menzel et al., 2018). This research topic focusses on the latest developments in the integrative pharmacology of L-arginine, L-citrulline and NO as modulators of cardiometabolic, kidney and lung disease as well as diabetes. L-arginine is a physiological and pharmacological agent with profound effects on health and disease. The references (Cziráki et al.; Huang et al.; Scott et al.; Shatanawi et al.) collected for this research topic demonstrate its decisive role and open up new perspectives for the research in the field. A dysregulation of L-arginine metabolism in the lung, airways and the respiratory tract can contribute to the development of acute and chronic lung diseases, such as infections of the respiratory tract, asthma, COPD, pulmonary hypertension and bronchopulmonary displasia (Shatanawi et al.). The use of natural arginase inhibitors such as L-citrulline increases L-arginine bioavailability to endothelial nitric oxide synthase (NOS) and thus improves NO formation for maintaining optimal cardiovascular antioxidant protection (Shatanawi et al.; Scott et al.). Current investigations to increase L-arginine availability to NOS, focusing on the provision of supplemental L-arginine and/or L-citrulline, as well as specific b vitamins such as folic acid to inhibit the competing enzyme, arginase, may lead to improvements in our understanding of the physiology and pharmacology of L-arginine and L-arginine-rich food.
